# Mortality rates and proximal causes of death in patients with Lewy body dementia versus Alzheimer's disease: A longitudinal study using secondary care mental health records

**DOI:** 10.1002/gps.5937

**Published:** 2023-05-19

**Authors:** Anne D. Kershenbaum, Annabel C. Price, Rudolf N. Cardinal, Shanquan Chen, James M. Fitzgerald, Jonathan Lewis, Sinéad Moylett, John T. O’Brien

**Affiliations:** ^1^ Cambridgeshire and Peterborough NHS Foundation Trust Fulbourn UK; ^2^ University of Cambridge Fulbourn UK; ^3^ Laboratory of Neuroimmunology KU Leuven Leuven Belgium

**Keywords:** cause of death, dementia groups, Lewy body dementia, mortality rates, secondary care mental health records

## Abstract

**Background:**

Previous studies have shown reduced survival in Lewy body dementia (LBD) compared to Alzheimer’s disease (AD), but the reasons for this are not known. We identified cause of death categories accounting for the reduced survival in LBD.

**Methods:**

We linked cohorts of patients with dementia with Lewy bodies (DLB), Parkinson’s disease dementia (PDD) and AD, with proximal cause of death data. We examined mortality by dementia group and hazard ratios for each death category by dementia group in males and females separately. In a specific focus on the dementia group with the highest mortality rate versus reference, we examined cumulative incidence to identify the main causes of death accounting for the excess deaths.

**Results:**

Hazard ratios for death were higher in PDD and DLB compared to AD, for both males and females. PDD males had the highest hazard ratio for death across the dementia comparison groups (HR 2.7, 95% CI 2.2–3.3). Compared with AD, hazard ratios for “nervous system” causes of death were significantly elevated in all LBD groups. Additional significant cause‐of‐death categories included aspiration pneumonia, genitourinary causes, other respiratory causes, circulatory and a “symptoms and signs” category in PDD males; other respiratory causes in DLB males; mental disorders in PDD females; and aspiration pneumonia, genitourinary and other respiratory causes in DLB females.

**Conclusion:**

Further research and cohort development is required to investigate differences by age group, to extend cohort follow‐up to the whole population and to investigate the risk‐balance of interventions which may differ by dementia group.

## Key points

- Survival is shorter in Lewy body dementia (LBD, referring to both Parkinson's disease dementia (PDD) and dementia with Lewy bodies (DLB)) compared with Alzheimer's disease (AD), but the reasons for this difference are not well established
- We identified cohorts of patients with dementia (male and female AD, PDD, and DLB dementia groups) referred into mental health services and linked to National Health Service (NHS) Hospital Episode Statistics and the Office for National Statistics to identify death dates and proximal cause of death
- Among patients with DLB and PDD compared to AD, those with PDD, especially males with PDD, had the highest hazard ratio for death. Aspiration pneumonia and nervous system causes of death accounted for a significant proportion of the excess deaths in the male PDD group compared to the male AD group
- Compared with AD, hazard ratios for nervous system causes of death were significantly elevated in all LBD groups. A range of cause‐of‐death categories were significantly more frequent across the LBD groups, with aspiration pneumonia, genitourinary causes and other respiratory causes elevated in more than one group

## 1 INTRODUCTION

It is known that survival from first presentation with cognitive impairment is shorter in Lewy body dementia (LBD)—referring to both Parkinson's disease dementia (PDD) and dementia with Lewy bodies (DLB)—compared with Alzheimer's disease (AD), but the reasons for this difference are not well established.^1^ In a metanalysis,^2^ non‐AD dementias were associated with worse survival compared to AD; however, causes of death were not reported. In a Swedish Dementia Registry study,^3^ increased hazard rates for death (relative to AD) were observed for all other types of dementia including frontotemporal dementia, Lewy Body dementia, vascular dementia and mixed dementia. In a previous study from our group, the time from first presentation with cognitive decline to death was significantly shorter in patients with LBD compared to AD.^1^ A number of demographic, clinical and temporal contributing factors were examined; however, a cause for the difference was not identified.

Possible reasons for this variation in survival in people with dementia include differences in co‐morbidities and levels of physical frailty. A number of physical problems may increase the risk of earlier death, such as reduced mobility and postural hypotension increasing falls; urinary and bowel dysfunction increasing urinary tract infections; and swallowing dysfunction increasing the risk of aspiration.^4^ Alternatively, or in interaction with physical vulnerabilities, variation in psychiatric symptoms and treatment might explain variation in mortality. In addition, some variation may be attributable to the pathology itself; certain dementia types may intrinsically progress more rapidly than others due to a more aggressive disease process.

In this study we focused on examining variability in survival by cause of death due to physical frailty or comorbid physical disorders. The LBD group is likely to have a higher burden of comorbid physical disorders than AD by the nature of the disease, such as autonomic dysfunction in DLB.^5^ In a Swedish registry study, a worse comorbidity profile in DLB patients compared with an AD group was found.^6^ Even within patients with AD alone, those with physical comorbidities have reduced survival. In a longitudinal follow‐up study of patients with AD in the US admitted between 1992 and 95 to care homes, increased mortality was related to advanced age, male sex, limitation in physical functioning, malnutrition, presence of pressure ulcers, a diagnosis of diabetes mellitus, and of cardiovascular diseases. These factors were independent predictors of mortality regardless of the severity of cognitive impairment.^7^

Here using secondary care electronic records from a single NHS Trust in the UK from patients referred into services 2005‐19, we compared survival and cause of death between the dementia types to identify cause of death categories accounting for the increased deaths in LBD versus AD.

## 2 METHODS

We identified cohorts of patients with dementia (AD, PDD, or DLB dementia groups) referred into mental health services of Cambridgeshire and Peterborough NHS Foundation Trust (CPFT) from 2005 to 2019. CPFT is a UK National Health Service (NHS) Trust providing secondary care mental health services plus some community services to its catchment area, a population of around 0.86 million. Using linked mortality data from NHS Hospital Episode Statistics and the Office for National Statistics, via NHS Digital,^8^ we identified death dates and proximal cause of death.

### 2.1 Approvals

The project has UK NHS Health Research Authority approval (reference 239236). Ethical approval was granted by the East of England Cambridge Central Research Ethics Committee (reference 18/EE/0029).

For transiently identifiable records linkage, approval was obtained from the UK Confidentiality Advisory Group (CAG reference 18/CAG/0015) under Section 251 of the UK National Health Service Act 2006. Data were de‐identified before researchers were given access (including blurring of dates of birth to month/year) and analysed under NHS Research Ethics approvals (reference 18/EE/0029). Patients and carers have been involved throughout the design of the process of cohort identification and data linkage that has allowed this analysis to take place. Patients and carers supported this use of de‐identified data for research purposes.

### 2.2 Identification of DLB and PDD cohorts in early and later study periods

We had previously identified a retrospective cohort of 251 LBD patients referred into CPFT from 2005 to 2012 (early study period)^1^ from CPFT electronic clinical records in the Care Records System systems of that time. The RiO data collection system (Servelec RiO electronic care record system) replaced these systems in 2013. Electronic record data from RiO and its predecessor systems are stored in a de‐identified form in the CPFT Research Database, which provides an arbitrary patient‐specific identifier (pseudonym).^9^ Between 2018 and 19, a further 660 patients with LBD were identified from the de‐identified RiO records from referrals into CPFT mental health services from 2013 onwards (later study period).

In both the early and later study periods, text searches for key word and phrases, and ICD codes (e.g. ‘Lewy’, ‘LBD’, ‘PDD’, ‘G31.83’) were used to identify potential cases of LBD (approx. 3000 potential cases). Manual case identification was used to identify true cases. Initially, the text records of these potential cases were checked by a study researcher to identify ‘true’ (e.g. “presenting with LBD”), ‘false’ (e.g. “does not have LBD”) and ‘maybe’ groups. The ‘maybe’ groups were further checked by two experienced clinicians with knowledge of dementia diagnostic criteria and symptom presentation to decide ‘true’ or ‘false’. Those finally identified as ‘true’ cases were included in the cohorts of LBD patients. Additionally, for both the early and later study periods, LBD patients were classified as PDD or DLB based on the timing of the cognitive impairment and the parkinsonian motor signs. For classification as DLB, the cognitive impairment was identified before or within 1 year of the motor signs, and for PDD the cognitive impairment was identified more than a year after the motor symptoms.^10^

### 2.3 Identification of AD comparison cohorts

A cohort of patients with AD referred into the service was identified in 2019 (Cohort construction details, Supplementary Figure S1). We included those with an ICD10 diagnosis code indicating AD (F00.1 or F001, dementia in AD with late onset). Other ICD10 codes, including those indicating vascular (F01) or mixed (F00.2) dementia, early onset dementia in AD (F00.0) as well as less specific codes dementia in AD (F00) and dementia in AD, unspecified (F00.9), were not included. Those in the cohorts of DLB or PDD but also appearing in the AD cohort remained in the DLB or PDD category because clinician judgement was used in preference to the RiO codes.

### 2.4 Identification of referral date as index date and measurement of follow‐up time

We extracted the first date of referral in the electronic record system for the early study period (patients referred to CPFT 2005–2012), and similarly the first date of referral in the RiO system for the later study period cohort (patients referred to CPFT 2013–2019). Referrals before 2005 for the early study period and before 2013 for the RiO system were excluded. Patients missing a referral date were excluded from analysis. The start of the follow‐up (index date) was taken as the date of first referral (rather than the diagnosis date) as referral date was more reliably recorded in the electronic records. Additionally, at referral date, most patients, although officially diagnosed after the referral date, were likely to have been experiencing symptoms at the point of referral, since they had been referred into services. The end of follow‐up was taken as the death date or the date of the end of the study (31/01/2020). The date of the end of the study was fixed as 31/01/2020 to avoid inclusion of the COVID‐19 pandemic period.

### 2.5 Identification of outcome, death and cause of death

Causes and dates of death were provided by from NHS Digital mortality data. We sent CPFT identifiers to NHS Digital for linkage (primarily based on NHS number) to data derived from Office of National Statistics (ONS) Mortality and Hospital Episode Statistics (HES) datasets.^8^ Death dates were available up to 2021. As described above, we followed up until 31/01/2020.

We used NHS Digital data to identify causes of death. Recorded causes of death derive from legally mandatory death certificates, which provide the proximal^11^ cause as well as underlying causes (either the disease or injury which initiated the train of events leading directly to death, or the circumstances of the accident or violence which produced the fatal injury). ONS Mortality data contains this information for all deaths registered in England and Wales.^11^ We used the proximal cause of death rather than underlying cause of death because we were interested in the disease or condition immediately leading to death.

We examined cause of death subgroups, using World Health Organization (WHO) International Classification of Diseases, Tenth Revision (ICD‐10) coding.^12^ Cause of death was coded into categories by ICD 10 code category: A‐B infections; C‐D.49 neoplasms; D.5‐D.9 haematological/immunological; E endocrine; F mental; G nervous system; H eye/ear: I circulatory; J other respiratory and J690 aspiration pneumonia (includes J690 but does not include codes J691 or J698); K digestive; L skin, M musculoskeletal, N genitourinary, O‐Q congenital/pregnancy related; R abnormal symptoms and signs; S‐T injuries; and U‐Z others. Respiratory deaths (J codes) were subdivided into aspiration pneumonia and other respiratory causes, because respiratory cause was the most common cause of death and also because aspiration pneumonia is a known mortality risk in Parkinson's disease.^13^ There were no deaths in the category “eye/ear”. A minority of deaths in the NHS Digital data provided were not associated with a cause of death, and classed as missing cause of death.

Supplementary Tables S1 and S2 show the ICD cause‐of‐death category in the study sample (1921 deaths) and the nervous system cause of death codes (*N* = 343) by dementia group, respectively.

### 2.6 Identifications of covariates age and sex

Age at referral was calculated using date of birth and date of referral into CPFT services. Sex was identified from demographic data.

### 2.7 Analysis

R was used for data preparation and statistical analysis^14^; the ‘survival’ package was used for analysis.^15^

Although our focus was on differences between dementia groups rather than sex differences, males and females were analysed separately because there are known sex differences in mortality rates and additionally there is evidence that PDD^16^ and DLB^17^ may manifest differently in males and females.

### 2.8 Mortality

**FIGURE 1:**
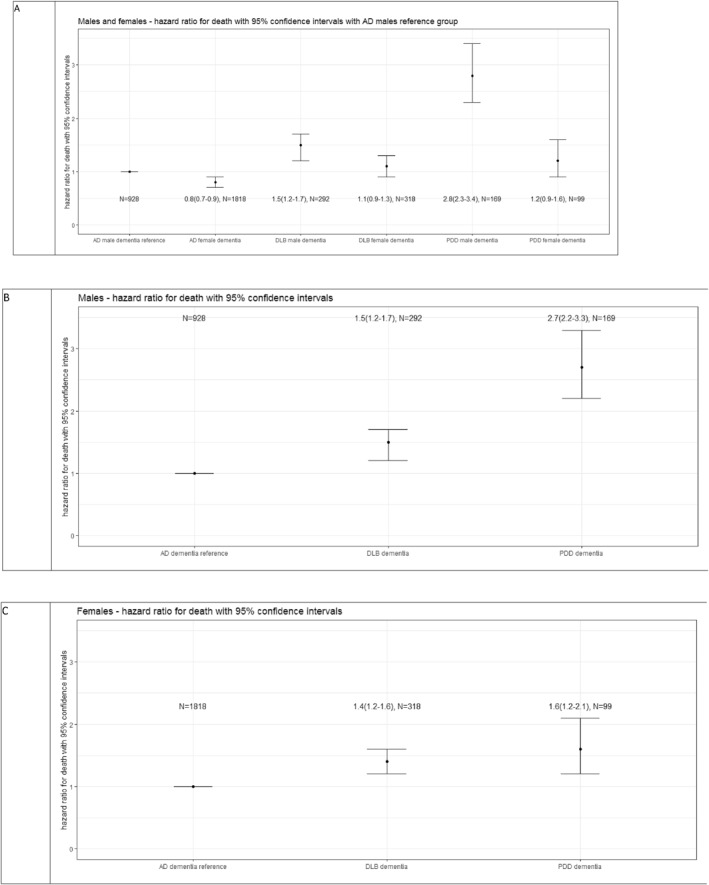
Hazard ratio for death by dementia group in patients with Parkinson's disease dementia (PDD), dementia with Lewy bodies (DLB), and Alzheimer's disease (AD). Upper panel (A) shows hazard rates for death with 95% confidence intervals for male and female dementia groups (reference group: AD males), middle panel (B) shows males only model (reference group: AD males) and lower panel (C) shows females only model (reference group: AD females).

We used Cox survival analysis to examine time to death from referral comparing the dementia groups (Figure 1A male and female dementia groups, Figure 1B males only dementia groups and Figure 1C females only dementia groups). We controlled for age at referral in Cox analysis. We used function ‘coxph’ from the *R* ‘survival’ package.

### 2.9 Hazard ratios by cause of death

We used competing outcome time‐to‐event analysis to compare ICD cause‐of‐death category in PDD and DLB groups versus AD (Table 2), in males and females separately. There is one event per person with 20 possible outcomes (no death; 18 observed categories of cause of death, as above; or death but missing cause of death.^18^ We used Cox proportional hazards (function “coxph” from R ‘survival’ packag*e*) where the event variable was treated as a factor and the first (reference) level of the factor was ‘no death’. The time of follow‐up was as described above. Categories F (mental disorders), R (abnormal symptoms and signs), and G (nervous system) were further examined at ICD‐10 code level to understand these proximal causes of death, where hazard ratios related to these categories were significant (Supplementary Table S2).

Examination of significant contributors to excess death in the dementia group with the highest hazard ratio for death.

**FIGURE 2:**
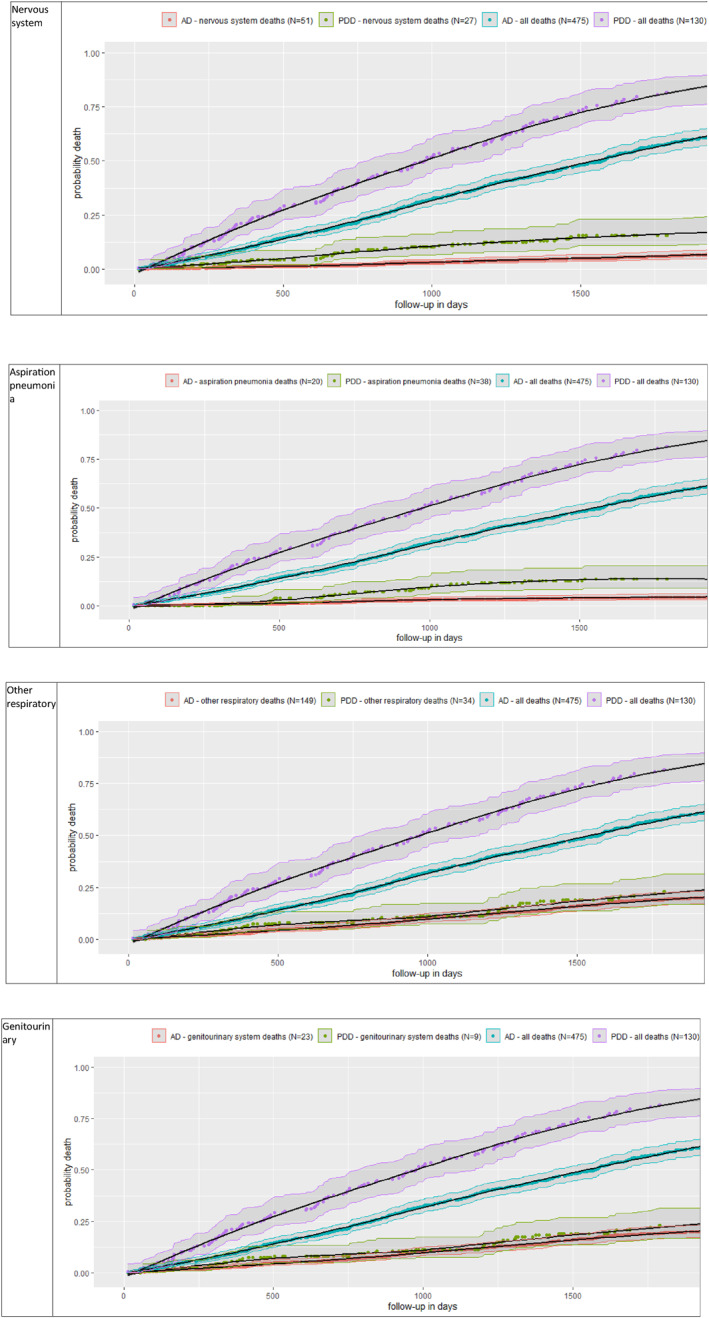
Cumulative probability of death for all causes of deaths, and the main cause‐of‐death categories: “nervous system”, aspiration pneumonia, “other respiratory”, “genitourinary” in males with Alzheimer's dementia (AD) compared to males with Parkinson's Disease Dementia (PDD). This specific comparison was examined because PDD males showed the highest hazard ratio for death versus reference (AD males). Smoothed black line (95% confidence interval shown in surrounding ribbon) shows cumulative probability of death.

We examined the main death categories contributing significantly to the excess deaths in the dementia group with the highest hazard ratio for death compared to the reference group We plotted cumulative incidences (equivalent to cumulative probability of death^18^) by cause of death category, using function ‘survfit’ from the R ‘survival’ package. We plotted from referral to death to maximum follow‐up time (Figure 2), along with 95% confidence intervals (CI). This analysis reports an absolute measure of effect^19^ to identify cause of death with most impact on the differences in mortality between the dementia group with the highest hazard ratio and the reference group. Figure 2 shows the cumulative probability of death (all causes) as well as cumulative probability of death by the individual death categories (each individual death categories shown in their own graphs). The difference between the cumulative probability of all‐cause death between the dementia category and the reference category (AD) at any given time represents the all‐cause gap. The more the corresponding gap for the individual death category approaches the all‐cause gap, the more that individual death category accounts for the excess deaths.

Sensitivity analysis ‐ analysis by study period, early or later (see above Identification of DLB and PDD cohorts in early and later study periods).

Hazard rates for death were calculated separately by study period, early or later, in the PDD and DLB dementia groups in males and females separately. This sub‐analysis examined whether hazard rates comparing early versus later study periods were similar and hence reasonable to combine by period (results shown in Supplementary Figure S2).

## 3 RESULTS

### 3.1 Descriptive statistics

In the study sample population of the AD group (*N* = 2746) versus the DLB and PDD groups separately (*N* = 610 and 268 respectively), the DLB and PDD groups were both younger (*p* < 0.001). There was also a higher proportion of females in the AD group (*p* < 0.001 for AD group vs. PDD and DLB groups separately (Table 1). In the PDD group 190/268 (71%) had died, versus 412/610 (68%) in the DLB group, and 1319/2746 (48%) in the AD group. This higher proportion of death in the PDD and DLB group versus AD is not unexpected as the PDD and DLB groups included those from the early study period (as well as the later study period).

**TABLE 1:** Cohort demographics (*N*, 3624) showing dementia groups (Alzheimer's (AD), Parkinson's Disease (PDD) and dementia in Lewy Body (DLB)).

*N* (total 3624)		AD	DLB	PDD
		2746	610	268
Sex (%)	Males,	928 (34%)	292 (48%)	169 (63%)
	*N* = 1389			
	Females	1818 (66%)	318 (52%)	99 (37%)
	*N* = 2235			
Age group, years (%)	65–75	403 (15%)	141 (23%)	76 (28%)
	75–85	1245 (45%)	274 (45%)	132 (49%)
	85–95	1007 (37%)	168 (28%)	47 (18%)
Median age at referral, years	Males	82	80	77
	Females	83	82	80
Death (%)	All, *N* = 1921	1319 (48%)	412 (68%)	190 (71%)
Death (%) by sex	Males	475 (36%)	193 (47%)	130 (68%)
	Females	844 (64%)	219 (53%)	60 (32%)

### 3.2 Hazard ratios for death

In each dementia type, males showed higher hazard ratios than females (though in DLB confidence limits just overlapped) (Figure 1 upper panel (A)). PDD males showed the highest hazard ratio for death compared to AD males (HR 2.7, CI 2.2–3.3 in a males‐only analysis, (Figure 1 middle panel (B)). PDD males showed a higher hazard rate for death than DLB males (HR 2.7, CI 2.2–3.3 vs. HR 1.5, CI 1.2–1.7 respectively, (Figure 1 middle panel (B)), but in females confidence intervals for PDD and DLB were overlapping (HR 1.6, CI 1.2–2.1 vs. HR 1.4, CI 1.2–1.6 respectively, (Figure 1 lower panel (C)).

### 3.3 Cause of death frequency

In males, ‘other respiratory deaths’ were the most common cause of death in all dementia types (AD, PDD and DLB), while in females ‘other respiratory’ deaths were most common in AD, but ‘nervous system’ deaths most common in PDD and DLB (Table 2).

**TABLE 2:** Cause of death in dementia groups: Alzheimer's disease (AD), Parkinson's disease dementia (PDD), and dementia with Lewy bodies (DLB).

Males											
	AD	DLB					PDD				
Cause of death	*N* = 928	*N* = *292*	HR	HR lower 95% CI	HR upper 95% CI	*p*	*N* = 169	HR	HR lower 95% CI	HR upper 95% CI	*p*
No death	453	99					39				
Infections	12	5	1.58	0.55	4.54	0.39	4	3.02	0.93	9.84	0.07
Neoplasms	27	9	1.00	0.43	2.28	0.99	4	1.32	0.45	3.92	0.61
Haematology/immune	0	0					0				
Endocrine	2	0					0				
Mental	58	21	1.27	0.75	2.15	0.37	9	1.71	0.83	3.53	0.15
Nervous system	51	37	**2.35**	**1.52**	**3.64**	**<0.01**	27	**4.42**	**2.70**	**7.24**	**<0.01**
Circulatory	52	14	0.95	0.52	1.75	0.88	12	**2.03**	**1.06**	**3.90**	**0.03**
Aspiration pneumonia	38	14	1.34	0.72	2.48	0.36	20	**4.55**	**2.57**	**8.05**	**<0.01**
Other respiratory	149	67	**1.65**	**1.22**	**2.22**	**<0.01**	34	**2.41**	**1.64**	**3.55**	**<0.01**
Digestive	8	3	1.32	0.34	5.08	0.68	1	0.90	0.11	7.51	0.92
Skin	1	0					0				
Genitourinary	23	8	1.37	0.61	3.09	0.45	9	**3.95**	**1.76**	**8.84**	**<0.01**
Congenital	1	0					0				
Symptoms/signs	45	9	0.72	0.33	1.57	0.41	7	**2.66**	**1.17**	**6.06**	**0.02**
Injuries	4	3	2.63	0.57	12.06	0.21	1	1.86	0.19	17.71	0.59
Others	0	0					1				
Missing cause	4	3	2.62	0.57	12.05	0.22	1	1.98	0.21	18.99	0.56

Females											
Cause of death	AD	DLB					PDD				
	*N* = 1818	*N* = 318	HR	HR lower 95% CI	HR upper 95% CI	*p*	*N* = 99	HR	HR lower 95% CI	HR upper 95% CI	*p*
No death	974	99					39				
Infections	21	6	1.73	0.70	4.29	0.24	2	2.16	0.50	9.28	0.30
Neoplasms	60	7	0.66	0.30	1.44	0.29	1	0.32	0.04	2.34	0.26
Haematology/immune	1	0					0				
Endocrine	2	1	2.56	0.23	28.75	0.45	0				
Mental	111	25	1.08	0.68	1.72	0.74	10	**2.02**	**1.05**	**3.90**	**0.04**
Nervous system	161	51	**1.63**	**1.17**	**2.26**	**<0.01**	16	**2.08**	**1.24**	**3.50**	**0.01**
Circulatory	108	27	1.32	0.85	2.04	0.21	4	0.78	0.29	2.14	0.64
Aspiration pneumonia	31	13	**2.33**	**1.21**	**4.50**	**0.01**	3	2.18	0.66	7.18	0.20
Other respiratory	185	50	**1.53**	**1.12**	**2.10**	**<0.01**	11	1.40	0.76	2.59	0.28
Digestive	19	4	1.04	0.32	3.40	0.94	1	1.17	0.15	8.98	0.88
Skin	3	0					0				
Genitourinary	15	9	**3.25**	**1.38**	**7.65**	**<0.01**	1	1.50	0.20	11.45	0.70
Congenital	0	0					0				
Symptoms/signs	103	21	1.07	0.66	1.75	0.78	7	2.05	0.94	4.46	0.07
Injuries	5	2	2.20	0.42	11.42	0.35	1	4.18	0.48	36.47	0.20
Others	1	0					0				
Missing cause	18	3	1.00	0.29	3.41	1.00	3	**3.93**	**1.14**	**13.51**	**0.03**

*Note*: Absolute numbers (N), hazard ratios (HR) and p values shown from multiple‐outcome Cox regression models, in which age at referral as well as dementia group included. Males and females analysed and shown separately. The rows with significant results are in bold.

### 3.4 Hazard ratios by cause of death

In males, hazard ratios for death attributed to nervous system disorders and other respiratory categories were significantly elevated in PDD and DLB relative to AD, and for PDD alone, aspiration pneumonia, circulatory, genitourinary and the “symptoms and signs” categories were also significantly higher. The highest hazard rate was associated with aspiration pneumonia in PDD (HR = 4.5 *p* < 0.01) (Table 2 males).

In females, hazard ratios for death attributed to nervous system disorders were significantly higher in PDD and DLB relative to AD. For DLB alone, aspiration pneumonia, other respiratory categories and genitourinary causes were also significantly higher and for PDD alone, mental disorders and missing causes were significantly higher. In PDD, hazard ratios for aspiration pneumonia and other respiratory categories were of similar magnitude to DLB females (HRs 2.3 and 1.5 respectively for DLB, and 2.2 and 1.4 respectively for PDD) (Table 2 females).

Nervous system cause‐of‐death codes (*N* = 343) are shown, by dementia type, in Supplementary Table S2 (in DLB 71/90 deaths were coded G318: Other specified degenerative diseases of nervous system while in PDD 31/47 deaths were coded G20: Parkinson disease). The “symptom and sign” category comprised 7 deaths in the male PDD group, 6 R54, Age‐related physical debility, and 1 R688, Other General Symptoms and Signs. The “mental disorder” category comprised 10 deaths in the PDD female group, 9 F03 and 1 F030, ICD codes for unspecified dementia.

### 3.5 Examination of significant contributors to excess death comparing PDD to AD in males (Figure 2)

The cumulative incidence 95% confidence intervals are non‐overlapping in categories aspiration pneumonia and nervous system deaths, and these two categories accounted for much of the gap between the cumulative incidence curves for PDD and AD total deaths.

### 3.6 Analysis by period, early or later

PDD males showed the highest mortality in both the early and later periods. HRs/CIs are shown in Supplementary Figure S2, adjusted for age at referral. Confidence intervals are overlapping comparing early and later periods in PDD males, DLB males, PDD females and DLB females and therefore early and later cohorts were combined.

## 4 DISCUSSION

Death rates were higher in PDD and DLB compared to AD in both males and females. Additionally, death rates were higher in males than females in all dementia groups. Examination of the main cause‐of‐death categories that accounted for the higher death rates in LBD (PDD and DLB) helps to identify the source of the higher death rates and direct potential preventative efforts. There was some similarity in death categories with elevated hazard ratios across the LBD dementia groups. Nervous system category deaths were elevated in all dementia groups versus AD. Since we already know that the LBD group have nervous system disease, this finding does not increase our insight into their increased death rates. However, respiratory death rates were elevated in PDD males and DLB females (both for aspiration pneumonia and other respiratory categories), and non‐aspiration respiratory deaths in DLB males. Additionally, genitourinary causes of death were elevated in PDD males and DLB females. LBD patients may be more prone to respiratory and genitourinary deaths presumably due to susceptibilities related to the neurological disorder. A number of other causes of death were elevated in specific dementia groups, such as cardiovascular and “symptoms and signs” categories in PDD males and mental disorders in PDD females.

Death rates were highest in PDD males and this finding directed our focus towards identification of the main causes of excess death in the PDD male group. Specific death categories could be associated with elevated hazard ratios but due to small numbers may not account for a large proportion of the excess deaths, and therefore an absolute method of analysis in addition to relative is helpful.^19^ We found that most of the excess deaths in PDD versus AD in males were accounted for by aspiration pneumonia or “nervous system” causes. The PDD group was younger than the AD group. Hazard ratios for death and for cause of death were adjusted for age. However, the examination of significant contributors to excess death comparing PDD to AD in males is based on cumulative incidences and not adjusted for age. If the PDD male group were older, the gap between the cumulative incidence curves for both PDD and AD total deaths and for individual death categories might be expected to be wider. This could potentially increase the number of death categories that show non‐overlapping cumulative incidence confidence intervals, to include categories such as genitourinary system deaths as well as nervous system deaths and aspiration pneumonia categories.

Comparing DLB to PDD, in males, hazard ratio for death was not as elevated in DLB, and in turn hazard ratio for aspiration pneumonia was not as elevated. This may represent a true lower risk for death in DLB compared to PDD in males, possibly due to less severe Parkinson's physical complications in our population of DLB patients. This would be unsurprising since by definition the cognitive decline is later in the progression of the disease in PDD compared to DLB, and physical symptoms are likely to be more severe or more progressed in PDD.^20^ However, in females, hazard ratios for death were similar in DLB versus PDD. The reason for this difference in the comparison of DLB to PDD between males and females is not clear but could represent a biological difference,^17^ difference in diagnosis, referral or treatment practices, or in coding tendencies when recording cause of death.

Most studies of mortality have found that AD has increased survival compared to PDD or DLB.^1^, ^3^, ^21^ However, PDD has not been associated with the highest mortality rates, as we found. Using Swedish registry data, Larsson et al.^22^ identified patients with DLB or PDD and found that higher mortality was associated with lower cognitive scores; however, there were no significant differences in survival between the dementia groups. As in our study, those patients had been referred into a secondary care memory clinic. Despite this similar set‐up, it is possible that this discrepancy is explained by referral of the most symptomatic PDD patients to our secondary care service while PDD patients with milder physical and cognitive symptoms may have been referred to neurology clinics or remained in primary care. If this is the case, survival would be expected to be associated with symptom severity or treatment rather than the dementia type itself. Further research based on the whole population is required to clarify the characteristics of the patients referred into services and to determine which symptom or treatment variables predict survival.

We encountered several issues related to cause of death. Underlying cause of death is often reported in the literature^11^, ^23^ complicating comparison to other studies. We collected the proximal cause of death rather than underlying cause of death because we were interested in the disease or condition immediately leading to death. Additionally, since we are using real‐world data, it is not unexpected that codes reflecting the dementia diagnosis that we are already aware of such as mental disorder, are recorded as the proximal cause of death. Such conditions, even though reported as the proximal cause of death, were less informative to the direct cause of death in our study. In contrast, categories such as aspiration pneumonia and genitourinary causes of death reflect mechanisms which are potentially influenced by treatment in a patient with dementia and potentially modifiable. In a recent study in patients with probable DLB enrolled from memory clinics in China, cause of death was as reported by the family or from medical records^24^ rather than ICD codes, with categories including failure to thrive (stopped eating or drinking) and multiorgan failure in addition to pneumonia or aspiration. Direct comparison of cause of death results with our study is therefore limited, due to a different assignment of death categories.

Pneumonia is the most common cause of death in both Parkinson's disease^25^ and in all‐cause dementia.^26^, ^27^ Using codes appearing in any place on the death certificate in a Swedish registry study, individuals with LBD had a higher risk of respiratory death than those with AD. The increased respiratory risk in LBD was hypothesized to be due to underlying parkinsonism.^28^ Autopsy results^26^ in patients with dementia showed bronchopneumonia was the commonest cause of death. In a recent nationwide (South Korean) database study,^13^ it was found that Parkinson's patients had four times the risk of aspiration pneumonia compared to matched controls and that approximately two‐thirds of the patients died within a year of the first occurrence of aspiration pneumonia. Additionally, dementia was found to be a significant risk factor for death due to aspiration pneumonia.

In the recent study in patients with probable DLB enrolled from memory clinics in China, use of anti‐psychotics predicted a shorter survival.^24^ Previous studies have identified cognitive impairment^29^ or dementia^30^ and symptoms of psychosis such as hallucinations^31^ in Parkinson's disease as risk factors for death. In a hospital‐based study^32^ of patients with Parkinson's disease admitted urgently to a neurology department, aspiration pneumonia was the most common reason for admission, and among the patients who were admitted due to aspiration pneumonia, most had cognitive impairment and/or a history of psychiatric symptoms. More research is required to clarify how the risk of aspiration could be reduced most effectively; potential interventions include careful assessment of swallowing function and review of feeding practices^33^ in high‐risk patients. There is some evidence that psychiatric medications might increase the risk of aspiration pneumonia. Use of antipsychotics has been associated with aspiration risk. Typical/atypical antipsychotic usage have been associated with 40%–50% increased odds of developing aspiration pneumonia during hospitalization in a non‐psychiatric population.^34^ Additionally, the use of antipsychotic drugs in elderly patients has been shown to be associated, dose‐dependently, with the risk of community‐acquired pneumonia.^35^ Possible mechanisms include sedation, a direct dopamine antagonist effect on dysphagia (as an extrapyramidal adverse reaction), or anticholinergic effects (which can impair swallow coordination).^36^ Benzodiazepine use is associated with an increased risk of pneumonia among patients with AD,^37^ plausibly because sedation increases the risk of aspiration. Agitation is a common psychiatric symptom in our patients and might itself be a risk factor for aspiration. Evidence for the impact of agitation on aspiration pneumonia in the literature is lacking, though we note that impulsivity and agitation are listed among the risk factors for aspiration pneumonia by the Canadian Patient Safety Institute.^38^ Further research on aspiration risk is warranted to disentangle the influence of medications prescribed and the indication for the prescription.

Hazard ratios for genitourinary deaths were also high in PDD (in comparison to both DLB and AD males) and were also found to be high in DLB females in comparison to AD females. Bladder dysfunction (urinary urgency/frequency) is a common nonmotor disorder in Parkinson's disease^39^ and in DLB.^40^ Antipsychotic medications may increase the risk of urinary tract infections due to anticholinergic activity.^41^ Other factors such as autonomic dysfunction and altered urodynamics, frailty and cognitive impairment, and the need for bladder catheterization are recognised as contributing to an increased risk of urinary tract infection in Parkinson's disease.^42^

Strengths of the present study include a large sample size of patients with DLB and PDD referred into psychiatry services, allowing direct comparison between these dementia subtypes. However, larger numbers would be required to investigate differences by age, and continued development of these cohorts over time is required. There are several limitations to this study. The cause of death as recorded by a clinician on the death certificate may not always reflect the actual, or most proximal, cause of death. Additionally, our study results relate only to patients referred into secondary care psychiatric services and may not reflect the population of those with dementia as a whole. It is possible that our finding of the highest hazard rate for death in PDD males may be due to referral into secondary care services of the most symptomatic patients in this group. However, these patients are most at risk of death in the population of patients we assessed and may be associated with specific vulnerabilities, potentially related either to symptoms or medications used in treatment. The study was based on real‐world data rather than data collected specifically for research. This has advantages such as good representation of the clinical setting and populations, but possible problems relating to data quality. For example, the codes used to identify AD cases (F00.1 or F001, dementia in Alzheimer's disease with late onset) were entered by clinicians. There is always a possibility of mis‐coding (e.g. recording F00.1 late‐onset Alzheimer's disease when the intention was F01 vascular dementia). Likewise, cause of death was taken from real‐world death certification, not always based on autopsy. It is possible that clinicians might be more likely to record aspiration pneumonia as a cause of death in a patient with PDD due to the known increased risk of aspiration in PDD. Additionally, we combined DLB and PDD cases referred during the early study period (2005 to 2012) with DLB and PDD cases referred in the later study period (2013 onwards), while the AD cases derived from the later study period only. Over this number of years, it is possible that there were changes in referral threshold or in treatment practices which might influence hazard ratios. However, confidence intervals of hazard ratios in DLB and PDD males and female groups, comparing early and later periods, overlapped.

## 5 CONCLUSION

Among patients with DLB and PDD compared to AD referred into secondary care psychiatry services, those with PDD, especially males with PDD, had the highest hazard ratio for death. Aspiration pneumonia accounted for a significant proportion of the excess deaths in the male PDD group compared to the male AD group. Further research is warranted to examine risk factors for aspiration pneumonia in patients with LBD referred into psychiatry services, particularly to clarify the balance of risks from medications in the treatment of symptoms such as psychosis and agitation. Other causes of death in LBD, such as genitourinary causes, also warrant further research to determine the risk/benefit ratio of medical interventions such as urinary catheterisation and treatment with antipsychotics.

## AUTHOR CONTRIBUTIONS

Anne D. Kershenbaum, Annabel C. Price, Sinéad Moylett, Shanquan Chen, Rudolf N. Cardinal and John T. O’Brien participated in the design of the study. Anne D. Kershenbaum, Sinéad Moylett, Jonathan Lewis and Annabel C. Price were responsible for cohort identification and data acquisition. Anne D. Kershenbaum and Shanquan Chen performed the data analysis and interpretation. Anne D. Kershenbaum, Rudolf N. Cardinal and John T. O’Brien drafted the manuscript. All authors critically evaluated the manuscript and gave their final approval before submission.

## CONFLICT OF INTEREST STATEMENT

The authors declare that there is no conflict of interest.

## Supporting information

Supporting Information S1

## Data Availability

Patient‐level data is not publicly available, under NHS Research Ethics terms. For details of researcher access to the CPFT Research Database, see https://www.cpft.nhs.uk/research‐database/. Source code and summary data are available on request.
