# Supplementary material for: Mortality rates and proximal causes of death in patients with Lewy body dementia versus Alzheimer's disease: A longitudinal study using secondary care mental health records
Source: Int J Geriatr Psychiatry. 2023 May 19;38(5):e5937. doi: 10.1002/gps.5937 (PMC10946736; doi:10.1002/gps.5937)
Supplement: Supplementary file 1 — Supporting Information S1 [file GPS-38-0-s001.docx]

**Supplementary Figure 1** - Cohort construction details

| **Tables linked to cohort table** | **Cohort table** |
| --- | --- |
| EARLY PERIOD TABLES- provide early period DLB and PDD groups (173 DLB and 78 PDD) identified from 2012 and demographic variables. Early period table was linked with early period referral table to get first referral date after 2005 | 24,772 patients - provides division into DLB/PDD/non DLB dementia/early period (DLB and PDD cases) and demographic variables  457 patients with DLB, and 203 with PDD from later period + 173 DLB and 78 PDD cases from early period, total 911 630 in DLB and 281 in PDD group  Of 2,812 with Alzheimer’s codes from diagnosis table, excluded 32 in DLB/PDD groups leaving 2,780 in AD group |
| RiO DIAGNOSIS TABLE from 2019 provides Alzheimer’s codes F00.1/F001 |  |
| RiO REFERRALS TABLE provides date of referral |  |
| RiO DEMOGRAPHY TABLE provides demographic variables |  |
| HES-ONS TABLE provides death, date of death and cause of death |  |

**Supplementary Table 1** – Cause of death for 3,624 patients, with 1,921 deaths

| ICD code | ICD group | N of total, 3624 |
| --- | --- | --- |
| A-B | infections | 50 |
| C-D49 | neoplasms | 108 |
| D50-60 | haematology/immune | 1 |
| E | endocrine | 5 |
| F | mental | 234 |
| G | nervous system | 343 |
| H | eye/ear | 0 |
| I | circulatory | 217 |
| J | respiratory | 496 |
| J69.0 | respiratory – aspiration pneumonia | 119 |
| K | digestive | 36 |
| L | skin | 4 |
| M | musculoskeletal | 0 |
| N | GU | 65 |
| O,P,Q | congenital | 1 |
| R | abnormal symptoms/signs | 192 |
| S-T | injuries | 16 |
| U-Z | others | 2 |
|  | missing | 32 |

**Supplementary Table 2**

ICD code details by specific death category (here shown without the dots in the ICD codes as appears in our data)

Nervous system cause of death codes (N=343) by dementia group

|  | AD | DLB | PDD |
| --- | --- | --- | --- |
| G122 | 1 | 0 | 0 |
| G20 | 0 | 6 | 31 |
| G200 | 1 | 0 | 4 |
| G219 | 1 | 0 | 0 |
| G231 | 0 | 2 | 0 |
| G238 | 0 | 1 | 0 |
| G301 | 2 | 0 | 0 |
| G308 | 1 | 0 | 0 |
| G309 | 201 | 5 | 1 |
| G318 | 2 | 71 | 6 |
| G419 | 1 | 0 | 1 |
| G459 | 0 | 1 | 0 |
| G700 | 1 | 0 | 0 |
| G931 | 0 | 1 | 0 |
| G936 | 1 | 3 | 4 |

.

**Supplementary Figure 2**. Hazard rates with 95% confidence intervals in dementia groups (AD, PDD and DLB) by early or later period (males and females shown separately where the reference group is AD males)).

| 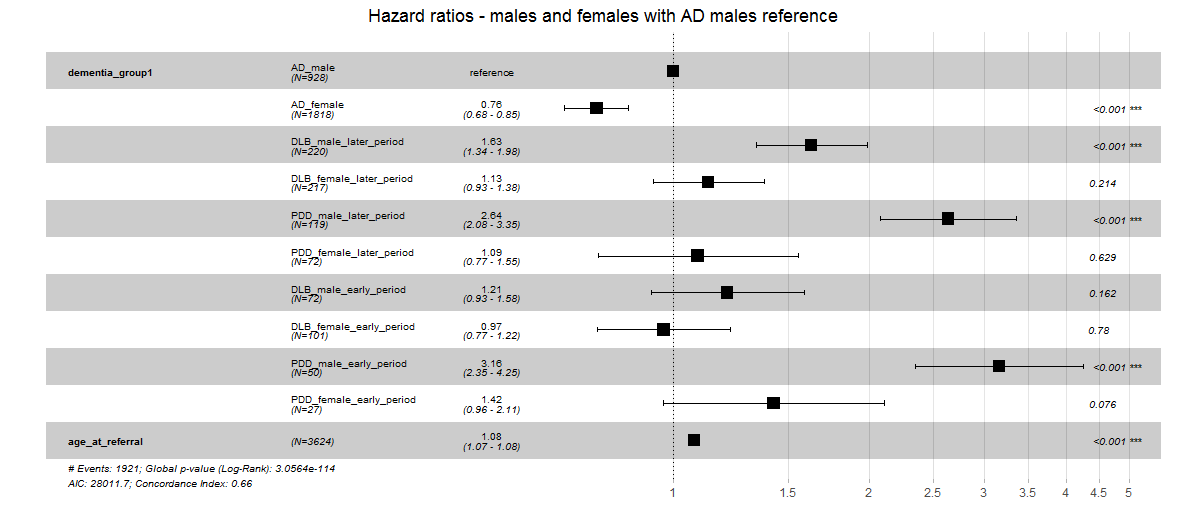 |
| --- |
